# The association between hydrochlorothiazide use and non-melanoma skin cancer in kidney transplant recipients

**DOI:** 10.1093/ckj/sfae126

**Published:** 2024-04-25

**Authors:** Ruth Rahamimov, Shay Telem, Batia Davidovichi, Dana Bielopolski, Tali Steinmetz, Eviatar Nesher, Shelly Lichtenberg, Benaya Rozen-Zvi

**Affiliations:** Department of Nephrology, Rabin Medical Center, Petah Tikva, Israel; Department of Organ Transplantation, Rabin Medical Center, Petah Tikva, Israel; Faculty l of Medical and Health Sciences , Tel Aviv University, Tel Aviv, Israel; The Maurice and Gabriela Goldschleger School of Dental Medicine, Tel Aviv University, Tel Aviv, Israel; Department of Dermatology, Rabin Medical Center, Petah Tikva, Israel; Department of Nephrology, Rabin Medical Center, Petah Tikva, Israel; Faculty l of Medical and Health Sciences , Tel Aviv University, Tel Aviv, Israel; Department of Nephrology, Rabin Medical Center, Petah Tikva, Israel; Faculty l of Medical and Health Sciences , Tel Aviv University, Tel Aviv, Israel; Department of Organ Transplantation, Rabin Medical Center, Petah Tikva, Israel; Faculty l of Medical and Health Sciences , Tel Aviv University, Tel Aviv, Israel; Department of Nephrology, Rabin Medical Center, Petah Tikva, Israel; Faculty l of Medical and Health Sciences , Tel Aviv University, Tel Aviv, Israel; Department of Nephrology, Rabin Medical Center, Petah Tikva, Israel; Faculty l of Medical and Health Sciences , Tel Aviv University, Tel Aviv, Israel

**Keywords:** basal cell carcinoma, hydrochlorothiazide, kidney transplantation, non-melanotic skin cancer, squamous cell carcinoma

## Abstract

**Background:**

hydrochlorothiazide (HCTZ) diuretics were correlated with an increased risk of non-melanoma skin cancer (NMSC) and melanoma in the general population. Information is a scarce regarding this effect in kidney transplant recipients who are at increased risk of skin malignancies under immunosuppression.

**Methods:**

Single-center retrospective analysis of adult kidney transplant recipients between 1 January 2010 and 31 December 2015. The primary outcome of the study was the first diagnosis of skin cancer that was removed and pathologically analyzed. Exposure to thiazides was defined as HCTZ use daily for at least one year at a dose of 12.5 mg.

**Results:**

Among 520 kidney transplant recipients, 50 (9.4%) were treated with HCTZ. During a median follow-up of 9.8 years, 67 patients underwent surgical removal and pathological analysis of at least one skin cancer. Exposure to HCTZ during the 3 years following transplantation was associated with an increased risk of skin cancer (*P* = 0.004). In a multivariate model, there was a significant association between HCTZ exposure and NMSC (HR 2.54, 95%CI 1.26–5.15, *P* = 0.007). There was a higher rate of basal cell carcinoma with HCTZ exposure, according to both univariate and multivariate analyses (HR 2.61, 95%CI 1.06–6.43, *P* = 0.037) and (HR 3.03, 95%CI 1.22–7.55, *P* = 0.017, respectively). However, no significant association was observed between HCTZ exposure and squamous cell carcinoma.

**Conclusions:**

These findings suggest a benefit of increased frequency of dermatologist inspection in kidney transplant recipients receiving HCTZ especially in increased ultraviolet exposure area.

## Graphical Abstract

**Graphical Abstract:**
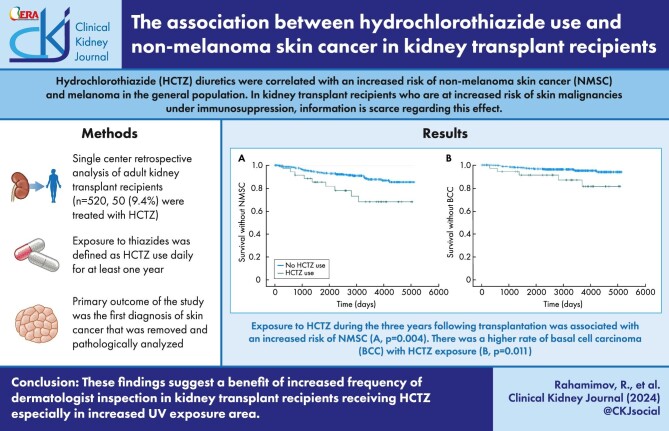

KEY LEARNING POINTS **What was known**:

- Kidney transplant recipients have a higher prevalence of hypertension mandating medication. They are at increased risk of developing non-melanotic skin cancers (NMSC) as a result of immunosuppression. Hydrochlorothiazide (HCTZ) diuretics have a photosensitivity effect and are associated with various types of skin malignancy in the general population. This effect is inconclusive in the kidney transplant population.

**This study adds**:

- Three years following kidney transplantation exposure to HCTZ is associated with an increased risk of skin malignancy (*P* = 0.004). There is a higher rate of BCC in a dose–response effect. HCTZ exposure was not associated with SCC either in univariate or multivariate analyses.

**Potential impact**:

- HCTZ increase the risk of NMSC in kidney transplant recipients, particularly BCC. Kidney transplanted patients who are treated by thiazides should be followed periodically by a dermatologist and alternative medications should be sought in the presence of additional risk factors for NMSC.

## INTRODUCTION

Hydrochlorothiazide (HCTZ) diuretics are recommended as the first-line treatment for essential hypertension [1]. In recent years, awareness has increased regarding the photosensitivity effects of HCTZ, potentially leading to skin malignancies.

Under ultraviolet light exposure, HCTZ compounds, including a sulfonamide moiety, become activated, leading to the generation of reactive oxygen species. This process triggers inflammation, cytotoxicity, and DNA damage [2]. Several studies have documented the association between HCTZ and various types of skin cancer, such as squamous cell carcinoma (SCC), basal cell carcinoma (BCC), carcinoma of the lip, malignant melanoma, and Merkel cell carcinoma. However, the results of these studies have been inconsistent, offering conflicting outcomes and providing evidence of low strength [2, 3, 4]. In a large cohort study involving 3870 patients treated with HCTZ, no significant correlation was found between HCTZ treatment and non-melanoma skin cancer (NMSC) [5]. Conversely, a recent meta-analysis found an association between HCTZ use and an increased risk of NMSC and melanoma in non-Asian countries [6]. These conflicting pieces of evidence are probably the result of diverse racial and geographic parameters contributing to the inconsistent effect of HCTZ on the risk of skin malignancies.

Hypertension is highly prevalent in kidney transplant recipients [7], parallel to an elevated risk of malignancies, including skin malignancies [8]. In a single-center study, HCTZ use was associated with an increased risk of SCC in kidney transplant recipients [9]. Considering the scarcity of data in this at-risk population, our objective was to assess the risk of NMSC in kidney transplant recipients exposed to high ultraviolet radiation.

## MATERIALS AND METHODS

### Patient population

This retrospective study used the Rabin Medical Center transplant registry patients' electronic records. We identified all adult kidney transplant recipients (age >18 years) who underwent transplantation between 1 January 2005 and 31 December 2010. These patients were continuously monitored by nephrologists at our kidney transplant recipient clinic including regular follow-ups at our transplant dermatology clinic, with at least one visit per year. Exclusion criteria included graft loss or death within the first year post-transplantation and diagnosis of NMSC within the first year post-transplantation. There were no consistent data regarding NMSC occurrence before the transplantation and skin phenotype beyond ancestral origin.

The study was approved by the Rabin Medical Center Ethics Committee and was conducted in accordance with the Declaration of Helsinki and the Declaration of Istanbul.

### Immunosuppression

Maintenance immunosuppression included tacrolimus [10], starting on postoperative day 1, at a dose of 0.15 mg/kg. The target trough levels for tacrolimus were 8–12 ng/ml during the first 3 months after transplantation and 6–8 ng/ml thereafter. The antiproliferative agents included mycophenolate mofetil [11], which was started on postoperative day 1 at a dose of 2.0 g/d and reduced to 1.5 g/d on day 14, and mycophenolate sodium [12], which was started on postoperative day 1 at a dose of 1440 mg/day and reduced to 1080 mg/day on day 14. All patients received perioperative intravenous corticosteroid therapy with methylprednisolone 500 mg on day 0, 250 mg on day 1, and 100 mg on day 2, followed by oral prednisone 20 mg/day tapered to 5 mg/d within 3 months.

Induction therapy consisted of one of the following: anti-IL-2 receptor antagonist basiliximab [13] on days 0 and 4 at a dosage of 20 mg intravenously or rabbit anti-thymocyte globulin [14] at a dosage of 1.0–1.5 mg/kg intravenously for 3 days, starting intra-operatively.

The conversion of mycophenolate mofetil or mycophenolic acid to mTOR inhibitors was performed on a case-by-case basis by a nephrologist following the diagnosis of SCC or other malignancies.

### HCTZ exposure

We examined the patients' electronic records and medication lists from the follow-up clinic to identify the use of thiazide diuretics, as a standalone therapy, or in combination with other medications. Since no other thiazide diuretics are available in Israel, hydrochlorothiazide (HCTZ) was the sole thiazide used by our patients. For the purpose of this study, exposure to HCTZ was defined as a minimum daily dose of 12.5 mg taken for at least 1 year. Total HCTZ exposure was calculated by multiplying the annual dose by the number of years and was reported as milligram-years. For the Kaplan–Meier survival analysis exposure was considered positive if HCTZ was prescribed within 3 years from transplantation for at least 1 year.

### Outcomes

The primary outcome of the study was the first diagnosis of skin malignancy that was removed and pathologically analyzed, including SCC, BCC, Bowen's disease, and basosquamous carcinoma. The follow-up time ended at the patient's death or the end of follow-up (14 November 2022). No malignant melanoma was diagnosed during the follow-up period.

### Statistical analysis

Continuous data are presented as mean ± standard deviation or median with interquartile range, whereas dichotomous data are presented as rates and percentages. A two-sample *t*-test was used for normally distributed data, whereas the Mann–Whitney *U*-test was used for non-normally distributed data. Differences in dichotomous variables were assessed using the *χ*^2^ test, and the Fisher's exact test was used when the sample size was small.

Kaplan–Meier survival curves were used for time-to-event analysis, and the significance of differences between the curves was assessed using the log-rank test. Exposure to HCTZ was defined as HCTZ use within the first year after transplantation, and the outcome was evaluated after a minimum of 3 years and continued beyond that time frame. Univariate and multivariate time-varying Cox proportional hazards models were used for univariate and multivariate analyses, respectively. The time-varying variable was exposure to HCTZ or the cumulative exposure to HCTZ. The proportionality of the hazards was evaluated by adding an interaction term for each variable over time and assessing the null hypothesis. For the sake of KM analysis, the first occurrence, either BCC or SCC was counted. For the sake of either NMSC separately, each type was counted for their own group.

For the multivariate analysis, a stepwise forward regression model was used with a *P* value of 0.05 for inclusion to adjust for factors associated with the outcome. In addition, donor age, diabetes mellitus, and delayed graft function were prospectively considered potential confounders due to their association with HCTZ exposure and were introduced into the model.

## RESULTS

Our initial cohort included 577 patients. Seven (1.2%) were younger than 18 years of age, and one patient (0.1%) had a diagnosis of skin malignancy within the first year; 49 patients (8.5%) had a follow-up period of <1 year. The final study cohort comprised 520 patients (90.1%). Patients exposed to HCTZ had a higher incidence of diabetes, delayed graft function, and received kidney donation from younger donors. The characteristics of the study cohort based on the HCTZ exposure status are summarized in Table 1.

**Table 1:** Characteristics of the study cohort participants according to HCTZ exposure status.

	All	HCTZ	No HCTZ	*P*
*N*	520	50	470	
male (%)	345 (66.3%)	38 (76%)	307 (65.3%)	.129
age	48.2 ± 14.6	48.2 ± 12.8	48.2 ± 14.8	.983
living donor (%)	288 (55.4%)	25 (50%)	263 (56%)	.42
donor age	43.1 ± 14.5	39 ± 14	43.6 ± 14.5	.035
DGF	113 (21.7%)	17 (34%)	96 (20.4%)	.027
European ancestry (%)	271 (52.1%)	23 (46%)	248 (52.8%)	.363
recurrent transplantation (%)	71 (13.7%)	6 (12%)	65 (13.8%)	.72
ATG for induction (%)	82 (15.8%)	9 (18%)	73 (15.5%)	.649
DM (%)	144 (27.7%)	20 (40%)	124 (26.4%)	.041
smoking	123 (23.7%)	13 (26%)	110 (23.4%)	.681
h/o cancer (%)	6 (1.2%)	0 (0%)	6 (1.3%)	.422

Abbreviations: DGF, delayed graft function; ATG, anti-thymocyte globulin; DM, diabetes mellitus.

During a median follow-up of 9.8 years [interquartile range (IQR) 5.7–12.7] a total of 67 patients underwent surgical removal and analysis of at least one skin malignancy. Among them, 33 patients (49.3%) were diagnosed with SCC, 17 (25.4%) with BCC, 13 (19.4%) with both BCC and SCC ( at different time points), one patient (1.5%) with Bowen's disease, one patient (1.5%) with basosquamous carcinoma, and two patients (3%) with unspecified skin tumors.

### HCTZ exposure and the risk of non-melanoma skin cancer

**Figure 1:**
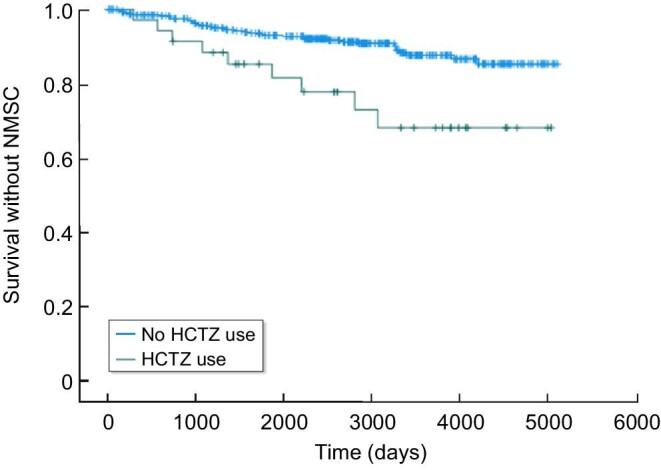
Kaplan–Meier NMSC-free survival curve, related to the use of HCTZ following kidney transplantation. HCTZ users (green) during the first 3 years following transplantation, vs. HCTZ nonusers (blue). *P* = 0.004.

Exposure to HCTZ up to 3 years following transplantation was associated with an increased risk of skin malignancy (*P* = 0.004) according to the Kaplan–Meier analysis (Fig. 1). In a univariate analysis, exposure to HCTZ was not associated with NMSC (HR 1.78, 95%CI 0.91–3.51, *P* = 0.095). However, in the multivariate adjusted model, the association became significant (HR 2.54, 95%CI 1.26–5.15, *P* = 0.007) (Table 2).

**Table 2:** Univariate and multivariate analyses for HCTZ exposure and the risk of NMSC.

	Univariate						Multivariate
Variable	HR	95% CI	*P*	HR		95% CI	*P*
diuretic exposure	1.781	0.905–3.508	.095	2.544		1.257–5.152	.009
male	0.700	0.408–1.201	.195		–	–	–
age	1.063	1.04–1.086	<.001	1.073		1.046–1.099	<.001
living donor	1.313	0.796–2.164	.286	1.850		1.078–3.176	.026
donor age	1.024	1.006–1.042	.008		1.017	0.998–1.037	.084
DGF	0.712	0.363–1.394	.322		0.615	0.298–1.269	.188
European ancestry	2.363	1.389–4.02	.002		2.723	1.593–4.655	<.001
recurrent transplantation	1.121	0.572–2.196	.739		–	–	–
ATG for induction	1.523	0.844–2.746	.162		2.186	1.174–4.07	.014
DM	1.473	0.878–2.472	.142		1.022	0.579–1.802	.941
smoking	1.167	0.663–2.054	.593		–	–	–
h/o cancer	1.614	0.224–11.653	.635		–	–	–

Abbreviations: DGF, delayed graft function; ATG, anti-thymocyte globulin; DM, diabetes mellitus.

Sensitivity analysis using only BCC and SCC definitive diagnoses (*n* = 63) yielded similar results in univariate and multivariate analyses (HR 1.96, 95%CI 0.99–3.88, *P* = 0.053 and HR 2.53, 95%CI 1.25–5.13, *P* = 0.01, respectively). Considering the cumulative dosage, a distinct correlation emerged between increased cumulative exposure to HCTZ and the incidence of NMSC. In univariate and multivariate analyses, respectively, these were HR 1.07/mg-years, 95%CI 1.01–1.13, *P* = 0.029 and HR 1.09/mg-years, 95%CI 1.03–1.16, *P* = 0.004.

### HCTZ exposure and the risk of specific skin malignancy types

**Figure 2:**
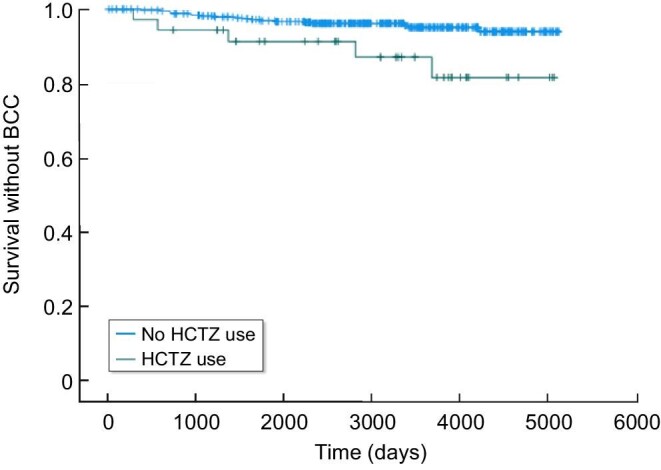
Kaplan–Meier BCC-free survival curve related to HCTZ use following kidney transplantation. HCTZ users (green) during the first 3 years following transplantation vs. nonusers (blue). *P* = 0.011.

Exposure to HCTZ during post-transplant was associated with an increased risk of BCC thereafter (*P* = 0.011), according to Kaplan–Meier analysis (Fig. 2). Exposure to HCTZ was associated with a higher rate of BCC in univariate (HR 2.61, 95%CI 1.06–6.43, *P* = 0.037) and multivariate analyses (HR 3.03, 95%CI 1.22–7.55, *P* = 0.017). The increased rate of BCC was also associated with higher cumulative doses of HCTZ (HR 1.006 per mg-years, 95%CI 1.001–1.012, *P* = 0.024) and (HR 1.007 per mg-years,95%CI 1.001–1.012, *P* = 0.012) in univariate and multivariate analyses, respectively.

**Figure 3:**
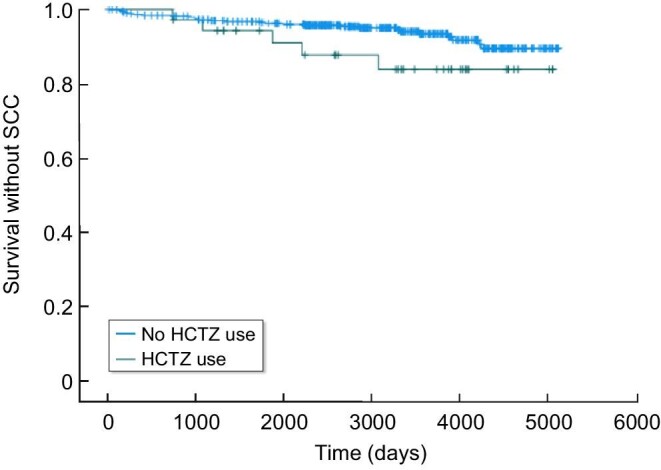
Kaplan–Meier SCC-free survival curve related to HCTZ use following kidney transplantation. HCTZ users (blue) during the first 3 years following transplantation vs. HCTZ nonusers (green). *P* = 0.105.

However, there was no association between HCTZ exposure and SCC [hazard ratio (HR) 1.3, 95%CI 0.51–3.32, *P* = 0.579] and (HR 1.57, 95%CI 0.61–4.04, *P* = 0.351) in univariate and multivariate analyses, respectively. In addition, exposure to HCTZ during the first 3 years was not associated with an increased risk of SCC according to Kaplan–Meier analysis (Fig. 3, *P* = 0.101).

## DISCUSSION

In a substantial cohort of kidney transplant patients with extended follow-up, we identified an increased risk of NMSC among those exposed to HCTZ in a dose–response manner. The heightened risk of skin malignancies linked to HCTZ exposure primarily stems from an increased incidence of BCC, rather than a significant association with SCC.

HCTZ stands as the most commonly prescribed diuretic in many European countries and the USA. However, both the European Medicines Agency and the US Food and Drug Administration (FDA) issued recommendations advising patients using HCTZ to be informed about the risk of NMSC and to undergo regular skin checkups [15]. An analysis of a UK dataset from general practitioners' questionnaires revealed that HCTZ users faced an increased risk of SCC compared to users of calcium channel blockers. Yet, no elevated risk was observed for BCC or malignant melanoma. Conversely, long-term indapamide use was associated with a higher risk of malignant melanoma but not SCC or BCC, whereas other diuretics did not pose additional risks for skin malignancies [15]. A recent meta-analysis linked HCTZ use to an increased risk of skin malignancies, especially SCC and melanoma, predominantly in non-Asian countries compared to other diuretics [6]. Multiple meta-analyses and cohort studies within the general population consistently indicate an association between HCTZ diuretics and NMSC development [16–19, 20]. However, different studies present varying risk patterns for BCC and SCC. Some studies suggest a higher risk of SCC than BCC, whereas others indicate an increased risk for SCC but not for BCC [21, 22].

Organ transplant recipients face a significantly higher risk of developing NMSC [23, 24]. The incidence of SCC is 250 times higher, and that of BCC is 10 times higher in organ transplant recipients compared to the general population. Moreover, the likelihood of developing the initial skin tumor rises exponentially with time [25]. Risk factors for the first episode of NMSC and its recurrence in kidney transplant recipients include older age, fair skin, and a history of NMSC before transplantation [26]. Intensive immunosuppression by tacrolimus appears to increase the risk of recurrence, whereas conversion to mTOR inhibitors reduces it, suggesting a mechanistic relationship [27].

To date, a solitary single-center investigation conducted in France by Letellier *et al.* has explored the correlation between exposure to HCTZ and NMSC in kidney transplant recipients. In contrast to our research, their results indicated a heightened occurrence of SCC among those exposed to HCTZ, while the risk of BCC did not show a significant increase [9]. Another discordance between the French study and ours lies in the proportion of BCC relative to SCC. In the French cohort, the ratio was ∼50% for each, with cumulative incidence rates of NMSC at 7% and 9% for SCC, and 8% and 11% for BCC after 10 and 15 years, respectively. In our study, 49.3% were diagnosed with SCC, 25.4% with BCC, and 19.4% with both BCC and SCC.

These discrepancies might be attributed to different skin types representing a variety of ancestries, different sun exposure related to geographical region, and the social behavior of suntanning causing recurrent acute photo-exposure (sunburns), which could influence the incidence and manifestation of different skin malignancies.

Our study has notable strengths, including its large cohort size and meticulous assessment of HCTZ exposure and skin cancer occurrence. However, it is essential to acknowledge several limitations. First, this is a single-center study, limiting the generalizability of findings to a broader population. Second, the study predominantly included individuals of Caucasian descent, cautioning against extrapolating these results to non-Caucasian populations. Third, is the missing data in our cohort regarding previous NMSC that could have added information to the NMSC risk analysis. Fourth, our study was not specifically designed to investigate the incidence of specific subtypes of skin cancer, potentially resulting in a type II error regarding the lack of association between HCTZ exposure and SCC. It is crucial to note that our study aimed primarily to investigate all types of NMSC rather than focusing on specific subtypes. The upper boundary of the confidence interval for the hazard of SCC ranged between 0.61 and 4.04 in our study. Last, the follow-up duration in our study was relatively short, while it is established that in many patients, NMSC tends to manifest in the second and third decades post-organ transplantation. Variations in follow-up times among our participants might contribute to disparities in study findings, particularly considering SCC and BCC may have distinct trajectories in their development and progression. Hence, long-term studies are warranted to comprehensively assess HCTZ exposure's effect on skin cancer risk and progression in this population.

In conclusion, our findings support an association between HCTZ exposure and the risk of NMSC in kidney transplant patients. Considering the notably increased baseline risk of NMSC in this population, caution is advised when prescribing HCTZ, taking into account the overall risk of skin cancer. These results underscore the importance of individualized risk assessment and monitoring strategies to mitigate the potential adverse effects of HCTZ on skin cancer risk in kidney transplant recipients.

## Data Availability

The data underlying this article will be shared on reasonable request to the corresponding author.
